# The role of netrin-1 in metastatic renal cell carcinoma treated with sunitinib

**DOI:** 10.18632/oncotarget.25201

**Published:** 2018-04-27

**Authors:** Sebastian Frees, Betty Zhou, Kyung Seok Han, Zheng Tan, Peter Raven, Alexander Wong, Ninadh D’Costa, Ladan Fazli, Werner Struss, Igor Moskalev, Claudia Chavez-Munoz, Alan So

**Affiliations:** ^1^ Department of Urologic Sciences, Vancouver Prostate Centre, University of British Columbia, Vancouver, Canada; ^2^ Department of Urology, University Medical Centre Mainz, Mainz, Germany

**Keywords:** renal cell carcinoma, sunitinib, resistance, netrin-1, tyrosine kinase inhibitor

## Abstract

**Introduction:**

Clear-cell renal cell carcinoma (ccRCC) is the sixth most common malignancy in men in North America. Since ccRCC is a malignancy dependent on neovascularization, current first line systemic therapies like sunitinib, target the formation of new vessels allowing nutrient deprivation and cell death. However, recent studies have shown that patients develop resistance after approximately 1 year of treatment and show disease progression while on therapy. Therefore, we propose to identify the protein(s) responsible for increased migration with the aim of developing a new therapy that will target the identified protein and potentially slow down the progression of the disease.

**Material and Methods:**

Human renal cancer cell lines (Caki-1, Caki-2, ACHN) were treated with increasing doses of sunitinib to develop a sunitinib-conditioned renal cell carcinoma cell line. mRNA microarray and qPCR were performed to compare the differences in gene expression between Caki-1 sunitinib-conditioned and non-conditioned cells. NTN1 was assessed in our *in vivo* sunitinib-conditioned mouse model using immunostaining. xCELLigence and scratch assays were used to evaluate migration and MTS was used to evaluate cell viability.

**Results:**

Human renal cell carcinoma sunitinib-conditioned cell lines showed upregulation of netrin-1 in microarray and q-PCR. Increased migration was demonstrated in Caki-1 sunitinib-conditioned cells when compared to the non-treated ones as well as, increased endothelial cell migration. Silencing of netrin-1 in sunitinib-conditioned Caki-1 cells did not demonstrate a significant reduction in cell migration.

**Conclusion:**

Netrin-1 is highly upregulated in renal cell carcinoma treated with sunitinib, but has no influence on cell viability or cell migration in metastatic RCC.

## INTRODUCTION

It is estimated that there will be 6,400 new cases of renal cell carcinoma (RCC) in Canada in 2016 and an estimated 1,850 patients will die from their disease [1, 2]. These deaths are mainly patients with metastatic disease progressing on targeted therapy. Despite the improvement made in response rates and progression free survival by the implementation of tyrosine kinase inhibitors into the standard care of metastatic RCC (mRCC), complete response and long term survival remains anecdotal and restricted to a limited number of patients [3, 4].

Despite treatment, patients with mRCC, once resistant, tend to have a fast increase in tumor burden locally and metastasize quickly. Sunitinib, a tyrosine kinase inhibitor, is one of the first line choices for the treatment of mRCC. Recent genetic analysis has emphasized the role of the VEGF-pathway in tumorgenesis and angiogenesis. As these mechanisms are so essential for the tumor, several studies have identified activation of alternative angiogenesis pathways as a possible mechanism of resistance. Despite this fact, several early clinical trials targeting those factors reported mixed results, therefore the quest for novel targets involved in the development of resistance continues.

Netrin-1, a laminin-related secreted protein, plays an important role in axonal guidance and development of neuronal and vascular structures during embryogenesis [5]. There is rising evidence that netrin-1 may also play a crucial role in carcinogenesis and metastasis in several cancer types through regaining its original involvement in angiogenesis in some cancer [6–9].

The need to elucidate potential biomarkers of early drug resistance and develop new agents to overcome treatment resistance in mRCC has led to the objective of this study, which is to evaluate the role of netrin-1 in mRCC cell lines (CAKI-1 and ACHN) after conditioning with the tyrosine kinase inhibitor sunitinib. If netrin-1 is the responsible factor for sunitinib resistance in mRCC, then we hypothesize that the downregulation of netrin-1 should result in decreased cancer cell migration and thus reduce disease progression in mRCC.

## RESULTS

### Metastatic renal cell carcinoma cells conditioned with sunitinib show an increased cell viability and migration

**Figure 1:**
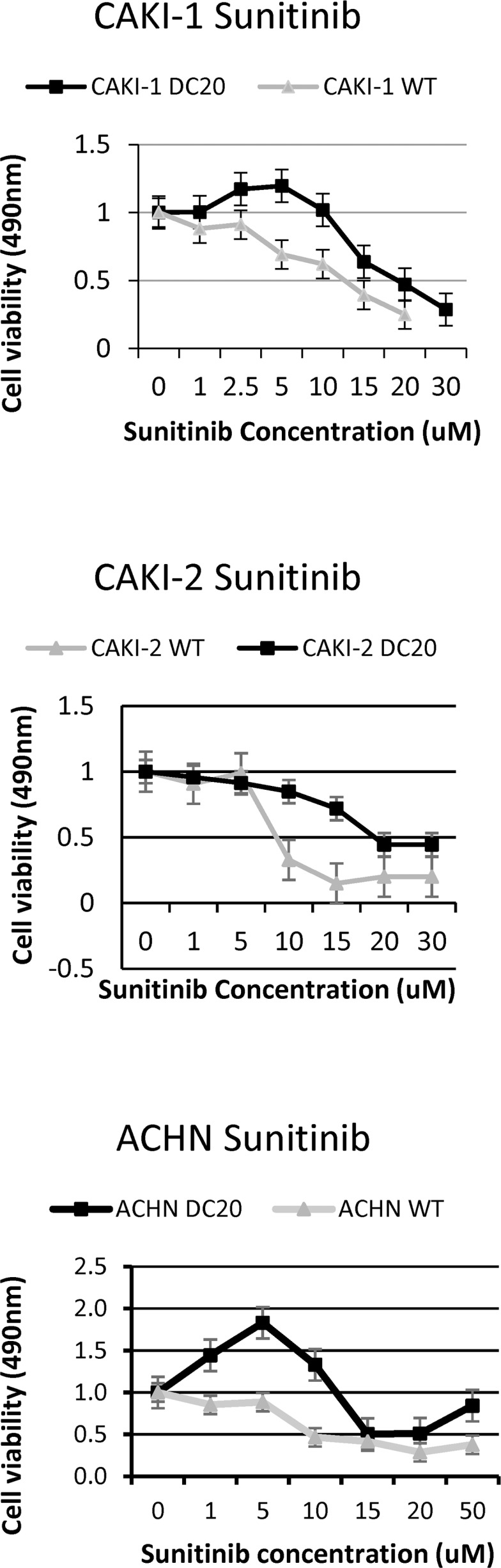
Increased tolerance of sunitinib conditioned RCC cell lines Sunitinib conditioned RCC cell lines (CAKI-1 DC20, CAKI-2 DC20 and ACHN DC20) show tolerance to higher doses of sunitinib compared to wildtype cells.

Renal cell carcinoma (RCC) cell lines (CAKI-1(mRCC), CAKI-2 (RCC) and ACHN (mRCC)) were grown and subjected to increasing doses of sunitinib (25% increments) from 0-22.5 μmol/L in order to create a RCC conditioned cell line. After approximately a couple of months, we were able to achieve a cell that would tolerate high doses of sunitinib treatment (max dose 22.5umol). Once established this, we calculated growth inhibition in order to compare wild type (WT) renal cell carcinoma cells (CAKI-1, CAKI-2 and ACHN) to the established drug conditioned cells. As shown in Figure 1, treatment with increasing doses of sunitinib revealed tolerance to the drug after time, increasing cell viability when compared to WT RCC cells.

**Figure 2:**
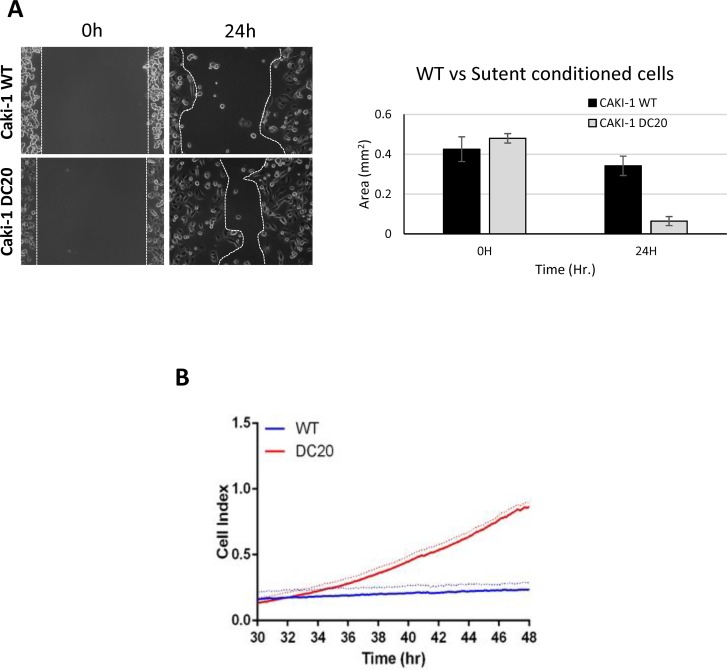
Sunitinib conditioned RCC cells show increase migration Increased migration was observed in CAKI-1 DC20 sunitinib conditioned cells using scratch assay (Caki-1 DC20 0.064mm^2^ ± 0.022 vs. Caki-1 WT 0.341mm^2^ ± 0.048; p=0.03) **(A)** and confirmed with Xcelligence **(B)**.

Furthermore, we performed scratch assays in RCC cell lines conditioned with sunitinib and WT (CAKI-1 Figure 2A, CAKI-2 Supplementary Figure 1) and measured gap area. After 24h, the results showed a significant increase in cell migration in the scratch assay for RCC sunitinib-conditioned cells, when compared to their corresponding WT group (0.064mm^2^ ± 0.022 vs. 0.341mm^2^ ± 0.048; p=0.03) (Figure 2A). These results were confirmed using xCELLigence migration analysis and expressed as a Cell Index value (Figure 2B). Based on these results we determined that RCC sunitinib-conditioned cells show increased migration capabilities than those from WT RCC cells.

**Figure 3:**
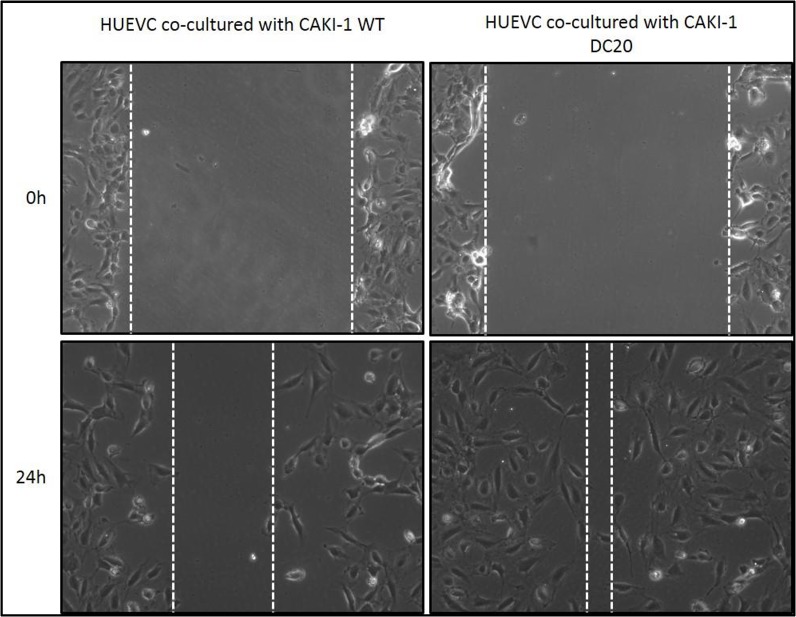
Scratch Assay of sunitinib conditioned RCC cells co-cultured with endothelial cells Increased migration was observed in HUVEC cells when co-cultured with CAKI-1 DC20 sunitinib conditioned cells using scratch assay.

Moreover, given the fact that sunitinib's mechanism of action is directly on endothelial cells by inhibiting tyrosine kinases, we wanted to evaluate migration on endothelial cells in a co-culture system with mRCC sunitinib-conditioned and WT cells. Results show that endothelial cells (HUVEC) significantly migrated faster when co-cultured with CAKI-1 sunitinib-conditioned cells than when co-cultured with RCC WT cells (Figure 3). These results may suggest that mRCC sunitinib-conditioned cells are capable of activating alternative proangiogenic pathways.

### NTN1 is highly upregulated in mRNA microarray and confirmed by PCR

**Figure 4:**
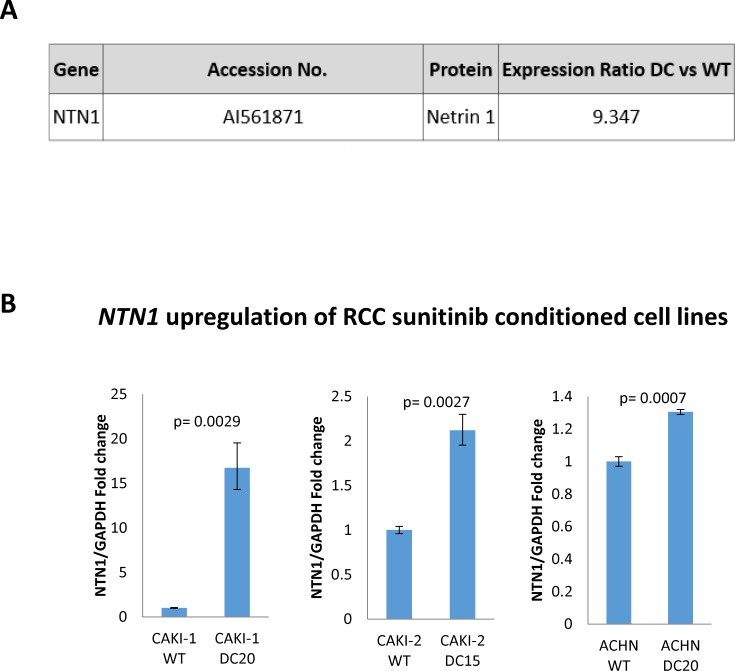
NTN1 Upregulation in sunitinib conditioned RCC cell lines **(A)** mRNA microarray showing upregulation of NTN1 in sunitinib conditioned CAKI-1 cells. **(B)** qPCR showing upregulation of NTN1 in three sunitinib conditioned RCC cell lines (CAKI-1 WT 1±0.040 vs DC2016.725±2.415; p=0.0029; CAKI-2 WT 1±0.0417 vs DC20 2.119±0.165; p= 0.0027 and ACHN WT 1±0.028 vs DC20 1.304±0.015; p=0.0007).

To follow with the results above, we decided to use a microarray to determine differences in gene expression between sunitinib –conditioned mRCC cells and WT mRCC cells. In this case and from now on we decided to use only CAKI-1 as mRCC representative cell line. Surprisingly, quantitative analysis of gene expression with microarray analysis revealed *NTN1* as one of the most upregulated genes in Caki-1 sunitinib-conditioned cells with a 9.347 fold increase when compared to WT (Figure 4A). In addition, qPCR confirmed a significant upregulation of *NTN1* in sunitinib-conditioned RCC cell lines (CAKI-1 WT 1±0.040 vs DC2016.725±2.415; p=0.0029; CAKI-2 WT 1±0.0417 vs DC20 2.119±0.165; p= 0.0027 and ACHN WT 1±0.028 vs DC20 1.304±0.015; p=0.0007) (Figure 4B).

### NTN1 is also highly expressed in a subcutaneous mRCC animal model when treated with sunitinib

**Figure 5:**
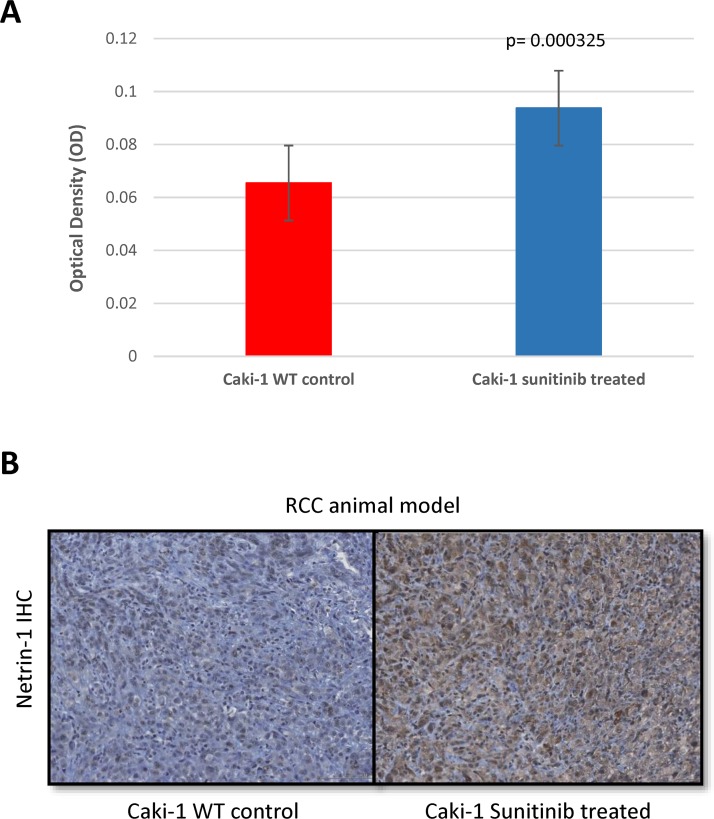
NTN1 upregulation in RCC animal model after resistance to sunitinib NTN-1 protein expression in Caki-1 xenograft mouse model in sunitinib-conditioned in comparison to sunitinib-sensitive mice tumors. **(A)** Uncalibrated optical density showing Caki-1 sunitinib treated 0.09 ± 0.002 n=39 vs Caki-1 WT 0.065± 0.003 n=26; p=0.0003. **(B)** Representative Images (10x magnification).

To expand on our findings, we decided to use our subcutaneous xenograft mouse model. We subcutaneously inoculated mRCC WT (CAKI-1) cells and once tumors reached 100mm^3^ the mice were randomly divided into 3 groups. One group was treated with sunitinib (40 mg/kg), the second group was treated with vehicle (citrate-buffered solution) and the third group was left untreated. Treatments were held for up to 48 days. After 48 days mice were sacrifized and tumors were subjected to immunohistochemistry and stained for NTN-1. Our findings demonstrate an increased expression in netrin-1 after treatment with sunitinib for 48 days when comparing to those of control (sunitinib-treated 0.093± 0.002 n=39 vs control 0.065± 0.003 n=26; p=0.0003) (Figure 5). These results confirm our in-vitro results showing a consistent increase expression of netrin-1 in mRCC cells when periodically exposed to sunitinib treatment.

### Silencing of NTN1 does not result in decreased migration or cell viability

**Figure 6:**
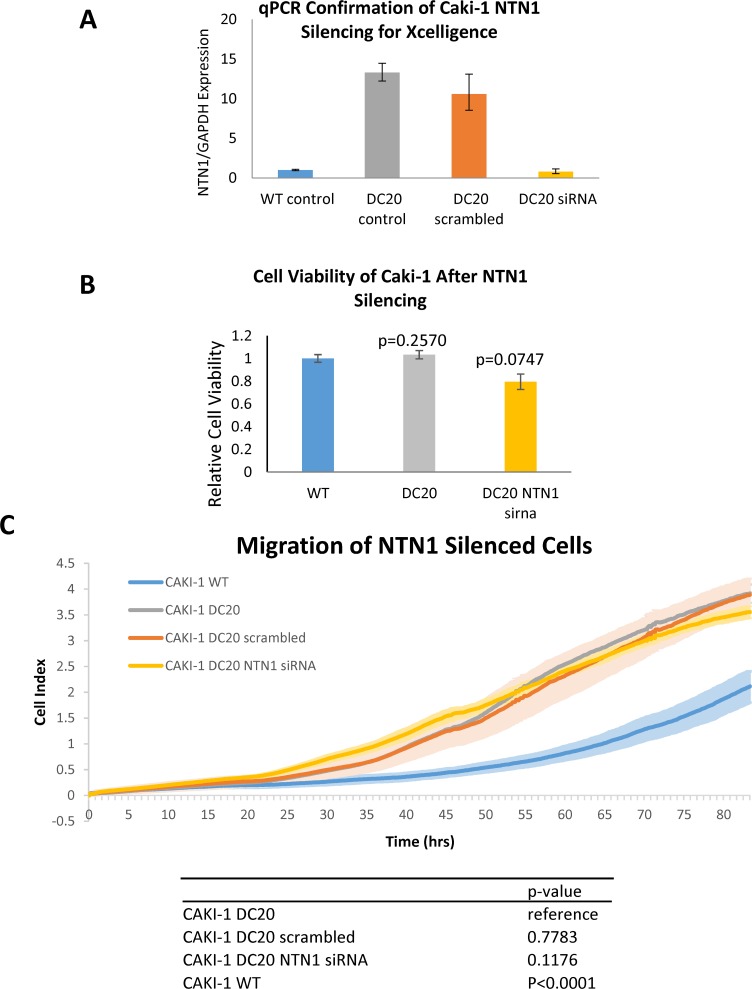
Migration of NTN1 silenced RCC cells **(A)** Downregulation of NTN1 in CAKI-1 DC20 cells silenced with NTN-1 siRNA was confirmed by qPCR prior to assessing the cells for migration using xCELLigence. **(B)** Cell viability between siRNA silenced and unsilenced cells was assessed to confirm that no significant difference in cell viability between Caki-1 WT, Caki-1 DC20 and Caki-1 DC20 NTN1 silenced cells was influencing migration results. (WT normalized to 1±0.033951 vs DC20 1.0333±0.0367 vs DC20 NTN1 sirna 0.79±0.0678, respectively with a p>0.05) **(C)** Xcelligence analysis shows that there is a significant difference in migration between Caki-1 WT and Caki-1 DC20 (p<0.0001), but no significant difference in migration between Caki-1 DC20, Caki-1 DC20 scrambled (p=0.7783) and Caki-1 DC20 NTN1 siRNA silenced (p=0.1176).

After seen consistent results of netrin-1 being overexpressed in mRCC sunitinib-conditioned cells, and also showing increased migration, we investigated the possibility of netrin-1 being directly responsible in increased cell migration. In other to achieve this, we started by silencing NTN-1 in CAKI-1 sunitinib-conditioned cells (CAKI-1 DC20). As shown in Figure 6A, specific silencing of NTN1 using siRNA resulted in a downregulation of NTN1, which was confirmed by qPCR. Silenced cells were evaluated for cell viability which could have influenced the results in migration. MTS was performed to confirm that silencing of NTN1 using siRNA did not have any significant change in cell viability (WT normalized to 1±0.033951 vs DC20 1.0333±0.0367 vs DC20 NTN1 sirna 0.79±0.0678, respectively with a p>0.05) (Figure 6B). Next step, was to evaluate migration in NTN-1 silenced CAKI-1 sunitinib-conditioned cells (CAKI-1 DC20). To achieve this, we performed scratch assays and xCELLigence migration analysis. As shown in Figure 6C, no significant difference in migration was observed between Caki-1 DC20 control, Caki-1 DC20 treated with scrambled siRNA (p=0.7783) and Caki-1 DC20 treated with NTN1 siRNA (p=0.1176). Therefore, we could not see an influence of *NTN1* knockdown on migration in Caki-1 sunitinib-conditioned cells, suggesting that netrin-1 has no effect in RCC sunitinib-conditioned cell migration.

## DISCUSSION

Sunitinib is the most commonly used first-line therapy for mRCC, unfortunately, 30% of patients are intrinsically resistant, while, 70% who initially respond will eventually acquire resistance [10]. This acquired resistance is demonstrated in our sunitinib-conditioned RCC cell lines, which show increased tolerance to sunitinib after each drug treatment cycle. The sunitinib-conditioned mRCC Caki-1 cells demonstrate increase migration, while also being able to increase endothelial cell migration when co-cultured with HUVEC cells, suggesting that sunitinib-condition mRCC cells are able to modify their migratory and angiogenic profiles to maintain adequate vasculature to proliferate even under VEGF inhibition. Such pro-migratory and angiogenic characteristics in resistant tumors treated with VEGF-targeted therapy has been extensively cited with the overexpression of anti-apoptotic factors like netrin-1 and angiogenic factors like FGF and Angiopoietin [11–13].

In our study, we observed *NTN1* to be highly upregulated in our in vitro sunitinib-conditioned mRCC cell lines as well as in our *in vivo* subcutaneous mRCC xenograft mouse model treated with sunitinib. Netrin-1 and its receptors, DCC and UNC5H, have been reported to be important factors for tumor development and progression in several tumor entities. Overexpression of netrin-1 has been associated with tumor progression in metastatic breast cancer [14], non-Hodgkin lymphoma [5] non-small cell lung cancer [15] and poorly differentiated pancreatic adenocarcinoma [16]. An imbalance in netrin-1 and DCC levels have been cited extensively, where the DCC chromosomal region have been reported to be frequently deleted in colorectal cancer, resulting in low DCC expression and thus cell survival. Netrin-1 overexpression has also been cited to increase the likelihood of colorectal tumor development [17, 18]. As a result, netrin-1 overexpression and/or the decrease in DCC or UNC5H have been described as a mechanism for cancer cells to inhibit proapoptotic signalling pathway, either through the autocrine loop of netrin-1, interaction of netrin-1 with its dependence receptor or through the absence of the dependence receptors, to promote cancer cell survival and thus increase cancer cell viability and migration [19].

Since the downregulation of netrin-1 has shown to reduce metastasis or tumor growth in multiple cancers, our study sought to investigate whether netrin-1 is also a promising target in mRCC. Fitamant et al. showed that the downregulation of netrin-1 by siRNA or the inhibition of netrin-1/dependence receptor interaction is able to promote apoptosis in tumor cell lines and prevent metastasis in both a mammary cancer cell line mouse model of lung colonization and lung metastasis-xenograft model of human breast cancer, but this reduction in metastasis was restricted to breast cancer cell lines or tumors expressing high levels of netrin-1 [14]. Similarly, Broutier et al. and Delloye-Bourgeois et al. have demonstrated anti-tumor effects in two aggressive subtypes of non-Hodgkin lymphoma and non-small cell lung cancer respectively through the interference of netrin-1 and its receptors in high netrin-1 expressing tumors [5, 15]. Unfortunately in our study, although we saw an upregulation of *NTN1* after drug conditioning with sunitinib in our mRCC cell lines and subcutaneous mouse model, we could not show a reduction in migration or cell viability in mRCC after NTN1 silencing. Therefore, suggesting that there are other factors other than netrin-1 that is responsible for metastatic disease in RCC.

In conclusion, this study demonstrates that netrin-1 is overexpressed in mRCC sunitinib-conditioned cells. However, migration of these cells were not affected by the presence or absence of netrin-1, but importantly, a significant difference in cell migration between mRCC sunitinib-conditioned and non-conditioned cell lines was observed. Our results show that netrin-1 may not be the responsible factor for progression in mRCC, but preliminary work suggests that other factors may be responsible. Future studies need to be performed to confirm the definite role of these factors in the development of resistance in mRCC in order to develop new therapeutics to slow down disease progression.

## MATERIALS AND METHODS

### Reagents

Sunitinib was used as a VEGF-targeted tyrosine kinase inhibitor. Free-base sunitinib and sunitinib malate were purchased from LC Laboratories (Woburn, MA). Mass spectrometry was used to ensure the quality of sunitinib when compared to pharmaceutical grade sunitinib (Pfizer, New York, NY). Free-base sunitinib was preserved as aliquots at a concentration of 10 mM in DMSO (Sigma St. Louis, MO) for *in vitro* experiments and sunitinib malate was mixed with citrate-buffered solution (pH 3.5) for *in vivo* studies.

### Cell culture

Human renal cell carcinoma cell lines: Caki-1(metastatic clear cell renal cell carcinoma), Caki-2 (primary clear cell renal cell carcinoma) and ACHN (metastatic papillary renal cell), were purchased from the American Type Culture Collection (ATCC Manassas, VA). Caki-1 and Caki-2 were maintained with McCoy's 5A medium (ATCC Manassas, VA) containing 10% Fetal Bovine Serum (FBS) (Gibco Grand Island, NY) and ACHN was maintained in RPMI 1640 medium (HyClone Logan, UT) containing 10% FBS and L-glutamine (Gibco Grand Island, NY). Human umbilical vascular endothelial cells (HUVEC) were also obtained from ATCC and maintained with EBM-2 PLUS medium (Lonza Basel, CH). Co-culture was performed using 6 well 3.0um pore sized PET membrane cell inserts (Corning New York, NY) and cells were co-cultured in a 50/50 mix of each individual Medium. All cells were cultured at 37°C in a humid atmosphere with 5% CO2. Mycoplasma contamination was tested every week.

### Establishment of sunitinib-conditioned tumor cells

Caki-1 cells were plated in 15-cm plates with McCoy's 5A medium with 10% FBS, grown to 50% confluence and incubated overnight for attachment. The cells were then exposed to sunitinib-containing media. The sunitinib concentration and exposure time were adjusted depending on the tolerance of the cells. Cells were exposed to sunitinib for 3–5 days and then replaced with fresh media without sunitinib for 24–48 h. Cells that showed proliferation at a specific sunitinib concentration were re-plated and exposed to a higher concentration (25% higher than the previous concentration). If the increased concentration was not tolerated, the cells were maintained in media with an identical or lower sunitinib concentration. The sunitinib on-off exposure cycle was maintained until the cells could proliferate in the presence of the target concentration (approximately 20 cycles). The final tolerated concentration of sunitinib was 22.5 μmol/L for Caki-1, 22.5 uM for ACHN, and 23.5uM for Caki-2. Cells were frozen at each cycle. Mycoplasma contamination was evaluated every 5 cycles.

### Cell viability assay using MTS

Cells were seeded onto 96-well plates at a density of 4 × 10^3^ cells per well in media with 10% FBS and allowed to attach for 24 h. DMSO or sunitinib was added at different concentrations. After 24 h of treatment, MTS reagent (Sigma-aldrich St Louis, MO) was added to cells (1:10 ratio) in each well and allowed to incubate at 37°C in a humid atmosphere with 5% CO2 for 2hrs. After that, plate was read at 490 nm using an Epoch spectrophotometer (BioTek Winooski, VT). All experiments were performed in triplicate and repeated a minimum of three times. Percent viability and cytotoxicity as well as standard deviations and IC50 were calculated from the absorbance values using Microsoft Excel.

### Scratch assay

Adequate amount of cells per cell type were plated to achieve a confluent monolayer in a 12 well plate. After treatment with 10ug/ml of Mitomycin C (Sigma-aldrich St. Louis, MO) for 2hrs, the cell monolayers were scraped in a straight line of similar size with a sterile p200 tip, debris and smooth edges were remove by washing the cells with 1 ml of PBS. Afterwards, PBS was replaced by the appropriate cell medium. Exact locations of each image were noted by the Axiovision microsope (Zeiss). Cells were incubated at 37°C for 24 hours depending on the migration rate of the specific cell type. Images were taken under a phase-contrast microscope matching reference coordinates. Image J software was used to analyse cell migration.

### Xcelligence

The migration of the wild-type (WT) and drug conditioned (DC) cell lines was measured using an xCELLigence system RTCA DP real-time cell analyzer (ACEA Biosciences Inc. San Diego, CA) fitted with CIM plates (05665817001, Roche Basel, CH).

These 16 well migration chambers consist of an upper and a lower chamber that are seperated by a porous polyethlene terephtalate (PET) membrane. The 8 um pores allow the cells to migrate from the upper chamber, where 2.0 × 10^4^ cells in FBS-free medium were seeded, to the lower chamber filled with media containing FBS. Every cell migrating to the lower chamber causes a change in the impedance of the xCELLigence system.

The analysis was performed every 15 minutes for the first 24 hours and once every hour thereafter. The experiments were repeated 3 independent times.

### mRNA microarray

Total RNA from the parental Caki-1 cells, and sunitinib-conditioned Caki-1 cells were obtained using mini RNeasy kit (Qiagen Hilden, DE). Three independent RNA samples were obtained from the cells to minimize experimental variation. Microarrays of 21,000 (70-mer) human oligonucleotides representing 21,000 genes (Operon Technologies) printed in duplicate in 3× SSC onto aminosilane-coated slides (ERIE C28) were used. Arrays were hybridized with 3DNA DNA Dendrimer Probes generated from 10 μg of total RNA according to the manufacturer's protocol (Genisphere Hatfield, PA). Briefly, reverse transcription incorporated a specific sequence present on the 5′ end of the reverse transcription primer supplied with the kit. The complementary DNA was hybridized to the array overnight at 42°C. After stringent washes, the fluorescent 3DNA reagent, which included a “capture sequence” complementary to the sequence at the 5′ end of the reverse transcription primer, was hybridized to the complementary DNA (47°C for 2-3 hr). After washing, the arrays were immediately scanned on a ScanArrayExpress Microarray Scanner (PerkinElmer Waltham, MA). Signal quality and quantity were assessed using Imagene 5.6 (BioDiscovery software El Segundo, CA). Data from Imagene were analyzed in GeneSpring 7.2 (Silicon Genetics Redwood city, CA) for profiling of changes in gene expression. Analyses performed in GeneSpring included background correction, LOWESS normalization and hierarchical clustering using standard correlation. Differentially expressed genes were identified by two-sample t-tests, and P values were adjusted for multiple comparisons using the false discovery rate method of Benjamini and Hochberg [20]. The Ingenuity Pathway Analysis (Ingenuity Systems, Mountain View, CA,) was used to examine functional and network associations between differentially expressed genes. Significances for biological functions were compared with the whole Ingenuity Pathway knowledge base as a reference set.

### Quantitative reverse transcription PCR (qPCR)

RNA was extracted from cell lines using RNeasy Mini Kit (Qiagen Hilden, DE), according to the manufacturer's instructions. RNA was quantified by the ratio of absorbance at 260/280 nm using a NanoDrop 2000 (Thermo Scientific Waltham, MA). First-strand cDNA was synthesized from 1-μg RNA by reverse transcriptase (Invitrogen Carlsbad, CA) with a random-hexamer primer. Taqman-primers used for qPCR included NTN1 (Hs00924151_m1) and GAPDH (Hs02786624_g1). Amplification was done using a Viia7 qPCR (Applied Biosystems, Foster City, CA). Target gene expression was normalized to GAPDH levels and the comparative cycle threshold (C_t_) method was used to calculate relative quantification of target mRNAs. Each assay was performed in triplicate with an *n* of 3-5 independent experiments.

### Tumor xenografts

All animal studies were performed in accordance to the guidelines of the Canadian Council on Animal Care (CCAC) with institutional certifications (University of British Columbia protocol No. A11-0385. Aseptic surgical techniques were used for all procedures. Parental Caki-1 cells (Caki-1-WT) (5 × 10^6^cells) were injected subcutaneously (SC) in the flank region of 8- to 10-week-old nude mice. When tumors reached a volume of 100 to 200 mm^3^, mice were randomized and divided into two groups (sunitinib versus vehicle treatment). Each treatment group consisted of eight mice. Sunitinib malate was suspended in citrate-buffered solution (pH 3.5). Tumor-bearing mice were orally administered (oral gavage) sunitinib malate (40 mg/kg) or vehicle (citrate-buffered solution) once daily for 48 days. Tumor volume was calculated using the equation tumor volume (mm^3^) = length × width × height × 0.5 and measured every 3 days using calipers. After 48 days, tumors were harvested, fixed in formaldehyde and paraffin embedded for immunohistochemical staining. All animals at the completion of the study were euthanized by inhalation of CO_2_ according to the animal ethics protocol.

### Immunohistochemistry

Following formalin fixation, tissues were embedded in paraffin. Sections (4um) were then prepared and mounted on slides for staining. Antigen retrival was performed using citrate buffer (pH 6.0) and steamed at high power for 17 min. Immunohistochemical staining of anti-NTN-1 (rabbit monoclonal [EPR5428] ab126729 Abcam Cambridge, UK) was achieved using the Ventana autostainer Discover XT (Ventana Medical Systems Oro Valley, AZ). Ultra-map DAB anti-Rb detection kit (760-151 Roche Basel, CH) was used as secondary antibody. Stained slides were digitalized with the SL801 autoloader and Leica SCN400 scanning system (Leica Microsystems Wetzlar, DE).

### Quantification mean count

All stained slides were digitalized with the SL801 autoloader and Leica SCN400 scanning system (Leica Microsystems; Concord, Ontario, Canada) at magnification equivalent to ×20. The images were subsequently stored in the SlidePath digital imaging hub (DIH; Leica Microsystems) of the Vancouver Prostate Centre. The digital images were analysed using FIJI Image J2 software (Open access University of Maddison-Wisconsin) and the values obtained (mean gray value) was converted into uncalibrated optical density (OD) using the formula OD = log (max pixel value/mean pixel value).

### Silencing NTN1 with siRNA

Caki-1 cells were cultured using standard procedures and silenced with either 10nm NTN1 siRNA (s18041Thermo Fischer Scientific, Waltham, MA) or 10nm scramble siRNA (4390843 Thermo Fischer Scientific). The transfection reagent used was Oligofectamine 2000 (Invitrogen Carlsbad, CA) and the transfection media used was Opti-MEM (Gibco Grand Island, NY). The siRNAs and oligofectamine were diluted in Opti-MEM and incubated for 20 mins at room temperature. Then the Opti-MEM mixture of siRNA and Oligofectamine was added to the cells and allowed to incubate at 37°C in a humid atmosphere with 5% CO2 for 24hrs. After 24hrs, the cells were boosted with another dose of siRNA treatment for 5 hours. After 48hrs, the cells were used for subsequent scratch assay, cell viability assay or harvested for RNA extraction.

### Statistical analysis

The Mann-Whitney U test was used to evaluate the statistical significance in the differences between groups. A P-value less than 0.05 was considered statistically significant.

## SUPPLEMENTARY MATERIALS FIGURE


