# Supplementary material for: The role of netrin-1 in metastatic renal cell carcinoma treated with sunitinib
Source: Oncotarget. 2018 Apr 27;9(32):22631–41. doi: 10.18632/oncotarget.25201 (PMC5978253; doi:10.18632/oncotarget.25201)
Supplement: Supplementary file 1 [file oncotarget-09-22631-s001.pdf]

# The role of netrin-1 in metastatic renal cell carcinoma treated with sunitinib

## SUPPLEMENTARY MATERIALS

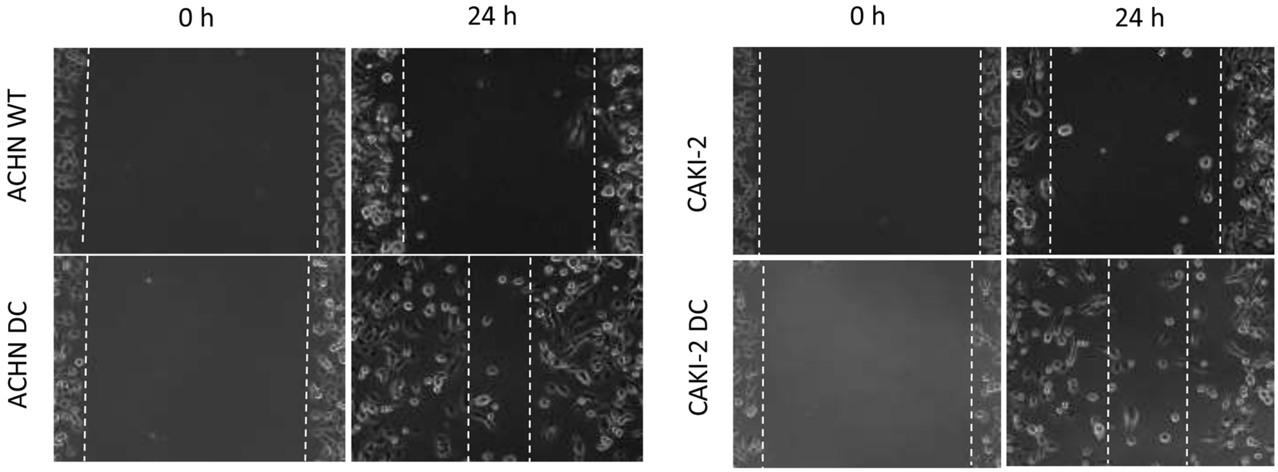


**Supplementary Figure 1: Sunitinib conditioned Renal cell carcinoma cell lines show increased migration.** Increased migration was observed in ACHN DC20 and CAKI-2 DC20 sunitinib-conditioned cells when compared to those of controls. A scratch assay was performed in cells previously incubated with Mitomycin C. Cells were visualized under the microscope at 0 and 24h. (Caki-2 DC20 0.444mm$^2$ ±0.12 vs. Caki-2 WT 1.202mm$^2$ ±0.054 p=0.001; ACHN DC20 0.534mm$^2$ ±0.014 vs ACHN WT 1.236mm$^2$ ±0.122; p=0.00129). Experiments done in three independent experiments. (Representative images).
